# Identifying patient subgroups in MASLD and MASH-associated fibrosis: molecular profiles and implications for drug development

**DOI:** 10.1038/s41598-024-74098-w

**Published:** 2024-10-07

**Authors:** Manuel A. González Hernández, Lars Verschuren, Martien P.M. Caspers, Martine C. Morrison, Jennifer Venhorst, Jelle T. van den Berg, Beatrice Coornaert, Roeland Hanemaaijer, Gerard J. P. van Westen

**Affiliations:** 1 Computational Drug Discovery, Leiden Academic Centre for Drug Research, Einsteinweg 55, 2333 CC Leiden, The Netherlands; 2 Unit Healthy Living and Work, TNO, The Netherlands Organization for Applied Scientific Research, 2333 BE Leiden, The Netherlands; 3 Galapagos NV, 2800 Mechelen, Belgium

**Keywords:** Liver disease, Heterogeneity, Patient stratification, Biological patterns, Individual variation, Subgroup-specific pathways, Cheminformatics, Computational biology and bioinformatics, Functional clustering, Machine learning, Experimental models of disease

## Abstract

The incidence of MASLD and MASH-associated fibrosis is rapidly increasing worldwide. Drug therapy is hampered by large patient variability and partial representation of human MASH fibrosis in preclinical models. Here, we investigated the mechanisms underlying patient heterogeneity using a discovery dataset and validated in distinct human transcriptomic datasets, to improve patient stratification and translation into subgroup specific patterns. Patient stratification was performed using weighted gene co-expression network analysis (WGCNA) in a large public transcriptomic discovery dataset (n = 216). Differential expression analysis was performed using DESeq2 to obtain differentially expressed genes (DEGs). Ingenuity Pathway analysis was used for functional annotation. The discovery dataset showed relevant fibrosis-related mechanisms representative of disease heterogeneity. Biological complexity embedded in genes signature was used to stratify discovery dataset into six subgroups of various sizes. Of note, subgroup-specific DEGs show differences in directionality in canonical pathways (e.g. Collagen biosynthesis, cytokine signaling) across subgroups. Finally, a multiclass classification model was trained and validated in two datasets. In summary, our work shows a potential alternative for patient population stratification based on heterogeneity in MASLD-MASH mechanisms. Future research is warranted to further characterize patient subgroups and identify protein targets for virtual screening and/or in vitro validation in preclinical models.

## Introduction

Metabolic dysfunction-associated steatotic liver disease (MASLD) is a prevalent chronic liver condition closely linked to the rise of obesity, metabolic syndrome, and type 2 diabetes mellitus^1^. MASLD is associated with hepatocellular damage, inflammation^2^ and fibrosis development^3^. Moreover, recent studies have identified liver fibrosis stage as an independent predictor of long-term mortality, regardless of other risk factors of MASLD or metabolic dysfunction-associated steatohepatitis (MASH)^4,5^. Despite intensive research to identify an anti-fibrotic drug, to our knowledge there is currently only one FDA approved drug (Resmetirom) in the market to combat liver fibrosis^6^.

Liver fibrosis, marked by the fibrous scar formation and tissue rigidity, is the result of the activation of hepatic stellate cells (HSC) into collagen-producing myofibroblasts, which increase extracellular matrix proteins deposition in the liver microenvironment. In the context of metabolic syndrome, MASLD manifests through increased de novo lipogenesis, lipotoxicity, glucotoxicity which in combination with hepatocellular damage, apoptosis, cytokine signaling and inflammation trigger repair mechanisms and liver fibrosis onset^7–10^. Considering the multifactorial pathogenesis of the disease, genetic predisposition, and environmental factors, there are multiple processes that can be deranged, thus highlighting the pathogenesis complexity and interindividual variability^11^.

To explore disease heterogeneity and unveil patient variability, recent studies have used omics datasets to identify patient subgroups both dependent^12^ and independent^13^ of disease severity. Undoubtedly, these approaches pave the way in deeper understanding of patient variability, nevertheless there are still big challenges for the drug development process especially to functionally annotate patient subgroups (gene and pathway level) and find representative preclinical models^14^. In line with this observation, preclinical models such as organoid systems recently highlighted that mechanistic insight is important to define anti fibrotic treatment^15^.

To better understand the large patient variability seen in MASLD-MASH on a molecular level, we used publicly available transcriptome data and pathology scores of individual patients to identify patient subgroups and characterize them on the pathway and gene-level. Subsequently, a predictive model that can classify unseen data into the distinct patient subgroups was trained. By stratifying MASLD^16^ or diabetic^17^ patients into subgroups that reflect the disease heterogeneity, a more realistic disease characterization of the patients and improved diagnosis can be achieved; thereby supporting therapeutic options and drug development.

## Methods

### Data preprocessing

Three public datasets from the GEO repository were re-analyzed, including GSE135251 (216 samples)^18^, GSE130970 (78 samples)^19^ and GSE240729 (55 samples)^20^. Each gene count data matrix was normalized relative to fibrosis stage 0 and log2 transformed (Rlog2). Inclusion and exclusion criteria and corresponding revisions by Ethical committees were specific for each study^18–20^.

### Weighted gene co-expression network analysis

The “WGCNA” package in R software was used to construct gene modules that are co-expressed (modules) in the discovery dataset GSE135251^21^. As explained above, the gene expression matrix was normalized and used as input to choose the optimal soft power threshold maximizing the scale-free network topology to generate gene modules (minimum 30 genes) based on hierarchical clustering method. Modules were refined by merging similar modules (those with a correlation ≥ 0.8 of their eigengene values).

Using the chooseTopHubInEachModule() function from the WGCNA package, hub genes were extracted as representative genes from each gene module. In the discovery dataset GSE135251, 15 gene modules were identified. The grey module was discarded as it contained uncorrelated genes. Using a matrix of 216 patients by 14 hub genes, hierarchical clustering was performed based on the Euclidean distance between rows and columns using the pheatmap function in R software^22^. The patient population was split into six patient subgroups independently of pathology scores (NAFLD associated score and Fibrosis score) based on literature reporting 3–8 patient subgroups^7,23–26^.

### Differential expression and pathway analysis

Log2fold change values of genes related to fibrosis stage (Fibrosis 4 vs Fibrosis 0) and change values of each patient subgroup versus the rest (e.g. Subgroup 1 vs Rest) were calculated using the DESEq2 package in R^27^. Genes were considered significantly differentially expressed (DEGs) if adjusted p-values were lower than 0.05. The extracted WGCNA gene modules, DEGs and important gene lists were analyzed using Ingenuity pathway analysis (IPA) for functional annotation on the pathway level and upstream regulators to obtain a mechanistic overview.

### Data augmentation techniques

As explained above, six patient subgroups were defined in the discovery dataset GSE135251 with varying group sizes. To define a predictive model data augmentation methods SMOTE (Synthetic Minority Oversampling Technique)^28^ and ADASYN (Adaptive Synthetic Sampling Approach)^29^ were used to improve the performance and generalizability of machine learning models in the six patient subgroups multiclass classification. The original dataset size (N = 216 samples) was divided into six patient subgroups 1–6 (57, 64, 46, 15, 27 and 7, respectively) as explained above. Data augmentation was applied to the training split (70%), using over-under sampling strategy 1 (SMOTE-1 and ADASYN-1, subgroups 1 = 25, 2 = 25, 3 = 25, 4 = 20, 5 = 20, 6 = 20) and over-under sampling strategy 2 (SMOTE-2 and ADASYN-2, subgroups 1 = 15, 2 = 15, 3 = 15, 4 = 15, 5 = 15, 6 = 15). Both SMOTE and ADASYN were used from the imbalanced-learn package in python^30^. Five training input datasets were evaluated to optimize 4 machine learning algorithms (random forest, decision trees, xgboost and k-nearest neighbors).

Performance was evaluated using nested cross validation (CV) with stratified inner (k = 2) and outer (n = 5) fold CV with the metrics Matthews correlation coefficient (MCC) and balanced accuracy (BA). Metrics were obtained 5*10 iterations resulting in 50 values per score for each model using the randomsearch() in scikit-learn^31^. ML models were fitted using a multiclass approach and 800 genes which were identified with a model-based feature selection approach. Model-based feature selection was obtained from statistical methods (multinomial logistic regression) using R^32^. For model refinement an extensive gridsearch was performed with dataset ADASYN-1 and the Random Forest algorithm for final model.

## Results

### Patient stratification

**Fig. 1:**
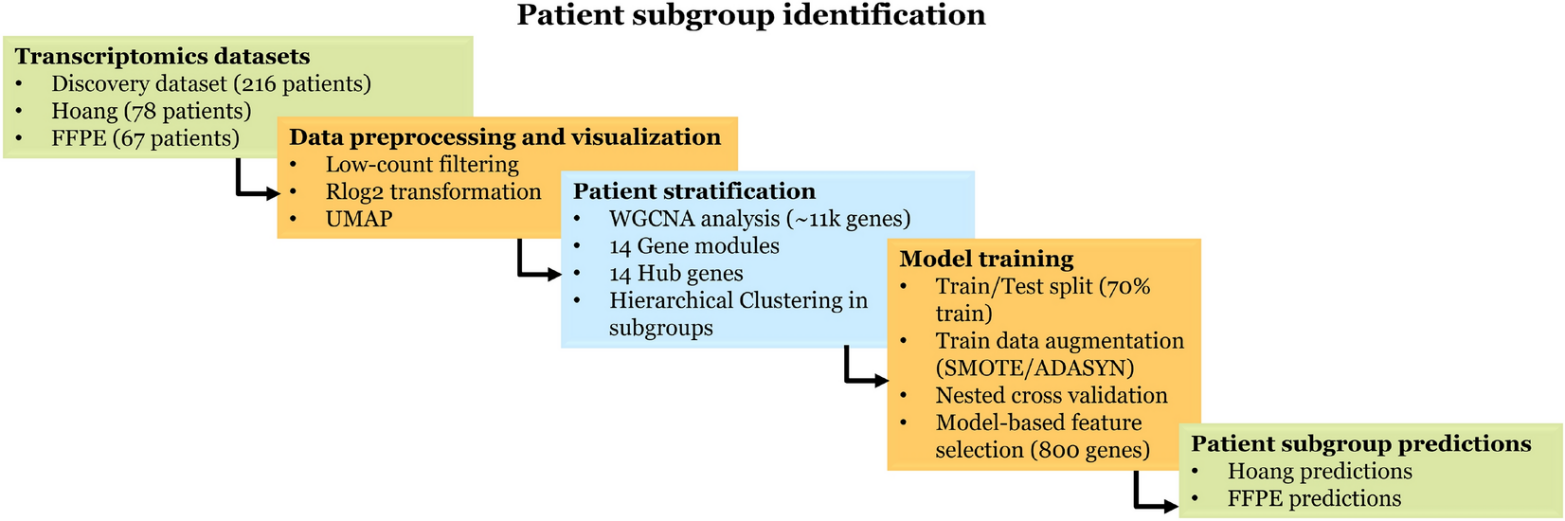
General workflow in the identification, characterization, and classifier construction of patient subgroups. Abbreviations: UMAP, Uniform Manifold Approximation and projection, WGCNA, weighted gene co-expression network analysis, SMOTE, Synthetic Minority Oversampling technique, ADASYN, Adaptive Synthetic Sampling Approach.

**Fig. 2:**
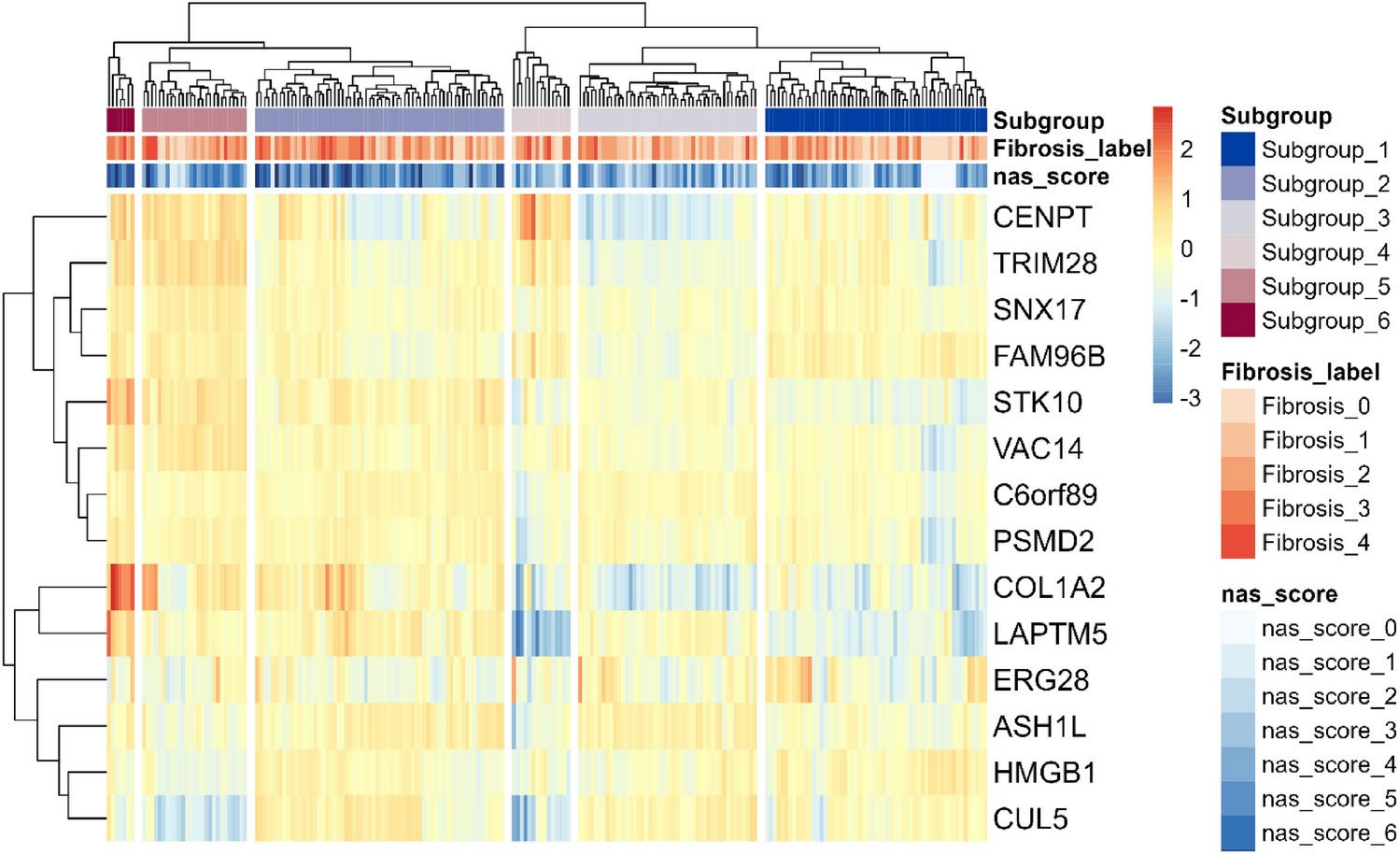
Patient stratification in the discovery dataset in 6 patients subgroups using 14 hub genes. The discovery dataset was stratified into 6 patient subgroups using 14 hub genes from gene modules. Hierarchical clustering was performed using Euclidean distance between rows (gene modules) and columns (patients). Each gene module is represented by the absolute expression of the corresponding hub gene. Subgroup 1 (n = #57), subgroup 2 (n = #64), subgroup 3 (n = #46), subgroup 4 (n = #15), subgroup 5 (n = #27) and subgroup 6 (n = #7).

WGCNA was applied to the discovery dataset to identify gene modules. This resulted in 14 gene modules of correlating genes independent of fibrosis staging. Using the 14 representative hub genes from the gene modules, the total patient population in the discovery dataset (216 samples) was clustered into six subgroups of different sizes using a hierarchical clustering method (See Figs. 1 and 2). Interestingly, these patient subgroups were defined independent of individual pathology scores (See Figs. 2 and 5, fibrosis label and NAS score), which might indicate that patient specific patterns are not completely related to disease severity (pathological scores). The patient distribution across the subgroups is as follows: Subgroup 1 (n# = 57), Subgroup 2 (n# = 64), Subgroup 3 (n# = 46), Subgroup 4 (n# = 15), Subgroup 5 (n# = 27), and Subgroup 6 (n# = 7) with their respective pathology scores (See Table 1). Subgroup-specific scores reveal minor variations across the patient subgroups which indicates no statistical differences of fibrosis and NAS scores per subgroup.

**Table 1:** Pathology score per patient subgroup.

Subgroup#	F-score [μ+ SD]	NAS score [μ+ SD]
Sub 1	1.42 ± 1.22	3.68 ± 2.21
Sub 2	1.45 ± 1.08	3.73 ± 1.55
Sub 3	1.93 ± 1.48	3.53 ± 1.64
Sub 4	1.98 ± 1.17	5.20 ± 1.70
Sub 5	1.85 ± 1.32	4.29 ± 1.87
Sub 6	2.85 ± 0.69	6.57 ± 0.97

Average pathology scores per patient subgroups. Abbreviations: F-score, Fibrosis score, NAS, NAFLD activity score.

### NAFLD heterogeneity

**Fig. 3:**
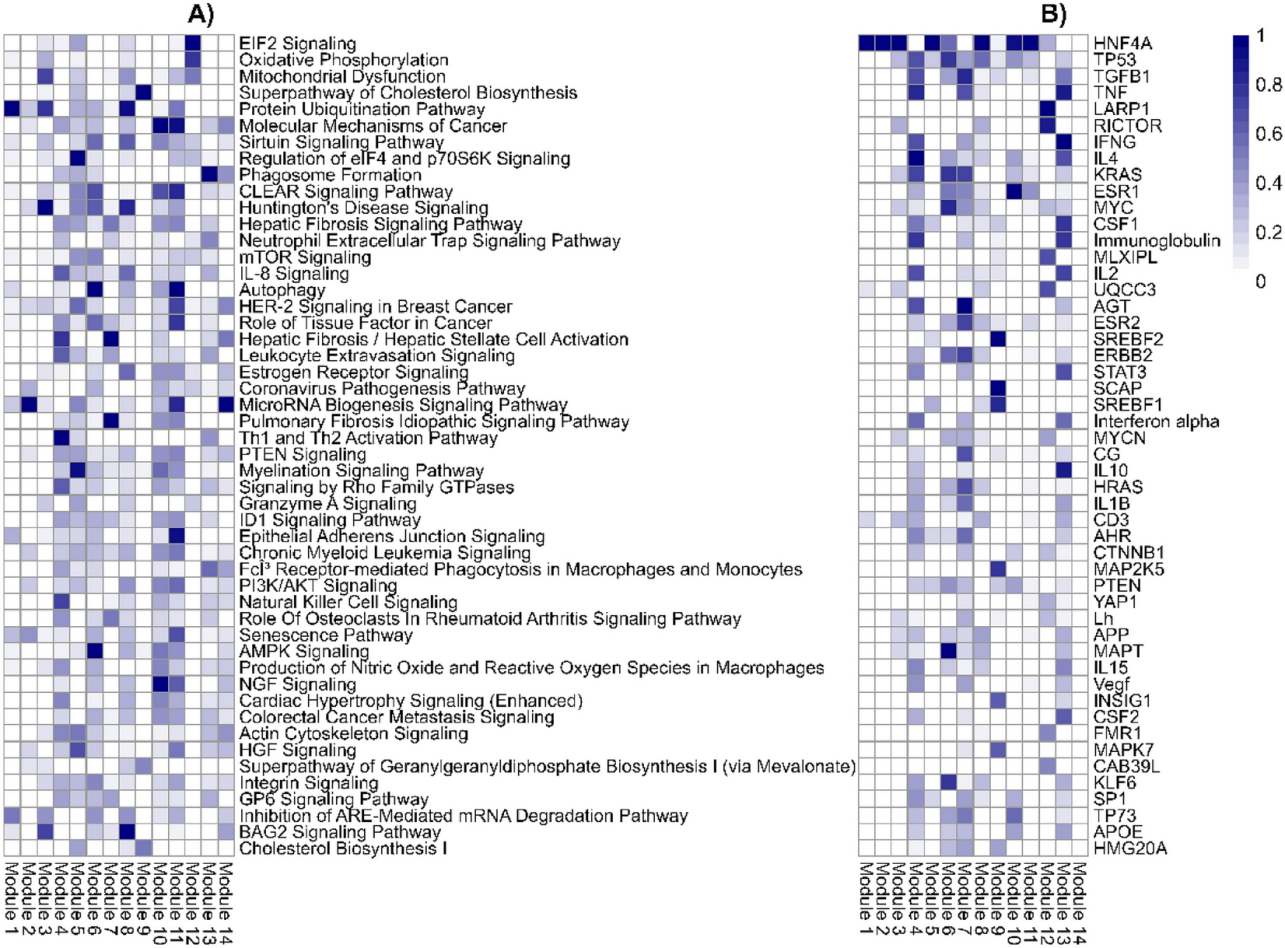
Canonical pathways and upstream regulators in 14 gene modules. (**A**) Top50 ranked canonical pathways and (**B**) Top 50 upstream regulators for each gene module from discovery dataset. Coloring indicates -logp-value (scaled 0–1). A higher enrichment corresponds with higher -logp-value.

**Fig. 4:**
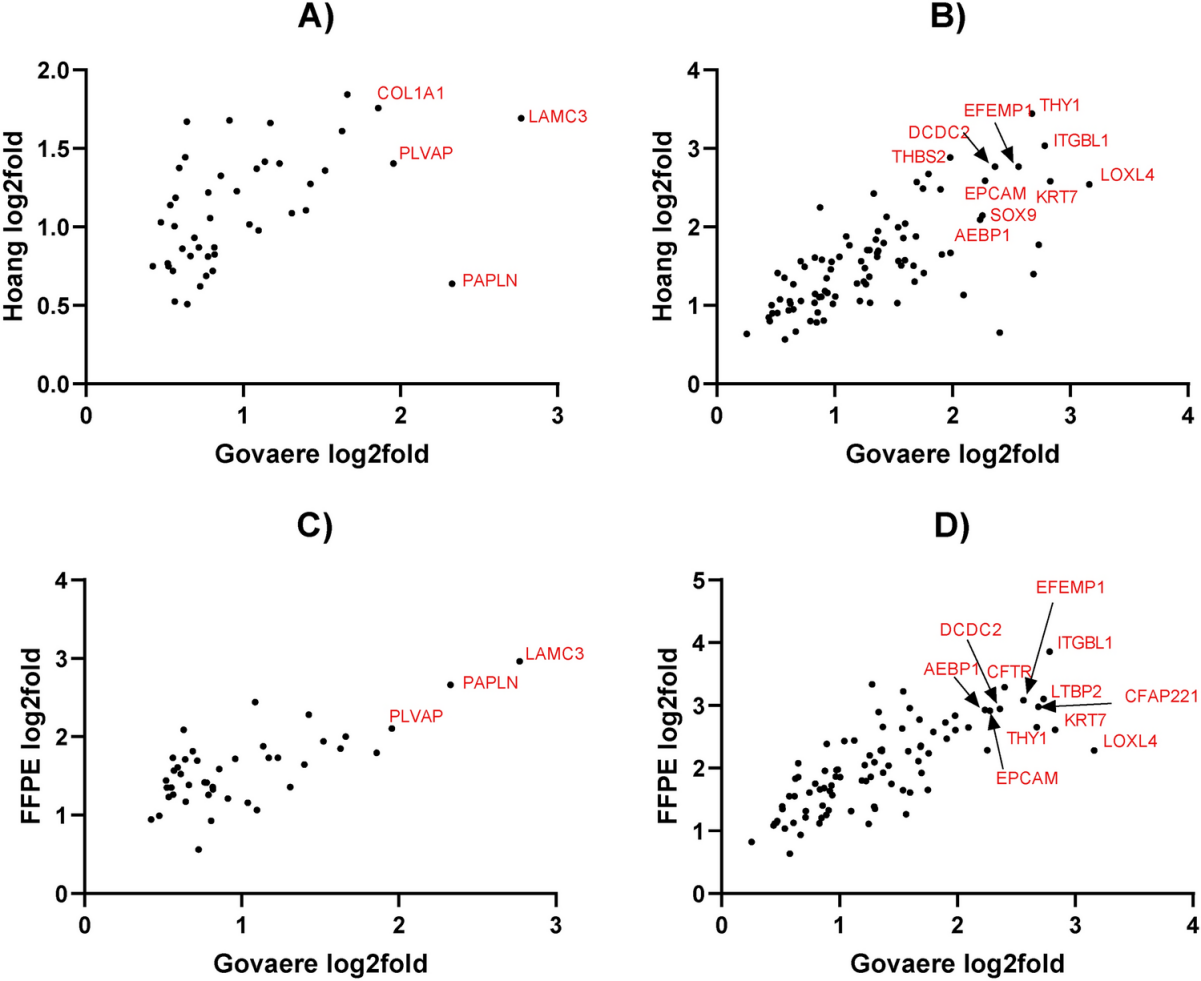
Directionality of genes in fibrotic gene modules 4 (448 genes) and 7 (260 genes) in the discovery dataset. Genes representing fibrotic core genes in clusters 4 and 7 were compared to differentially expressed genes (DEGs) shared in the three datasets (F4 vs F0 fibrosis scores, 246) including the discovery dataset and Hoang/FFPE datasets. Module 4 and Module 7 contain 44 and 90 DEGs, respectively.

The question arises whether various relevant MASLD-MASH related mechanisms and upstream regulators can be linked to the 14 gene modules. To answer this question, an Ingenuity pathway analysis was applied. Interestingly, this tool showed highly relevant liver-pathology-related processes such as cholesterol metabolism, immune pathways, extracellular matrix processes among other processes such as Eukaryotic initiation factor (EIF) signaling and mitochondrial dysfunction, indeed linked to these 14 gene modules (See Fig. 3). Of note, various modules have well-defined pathogenesis-related pathways and respective upstream regulators such as module 9 on cholesterol pathway and upstream regulator SREBF1. Additionally, modules relate to immune mechanisms and fibrosis mechanisms. First, modules 4 and 13 show immune mechanisms (Th1–Th2 pathway, IL-8 signaling) and immune regulators (TNF, IFNG, CSF1, IL-10, IL-4, STAT3). Second, modules 4 and 7 show hepatic fibrosis signaling and upstream regulator TGFβ1 (Fig. 4).

**Fig. 5:**
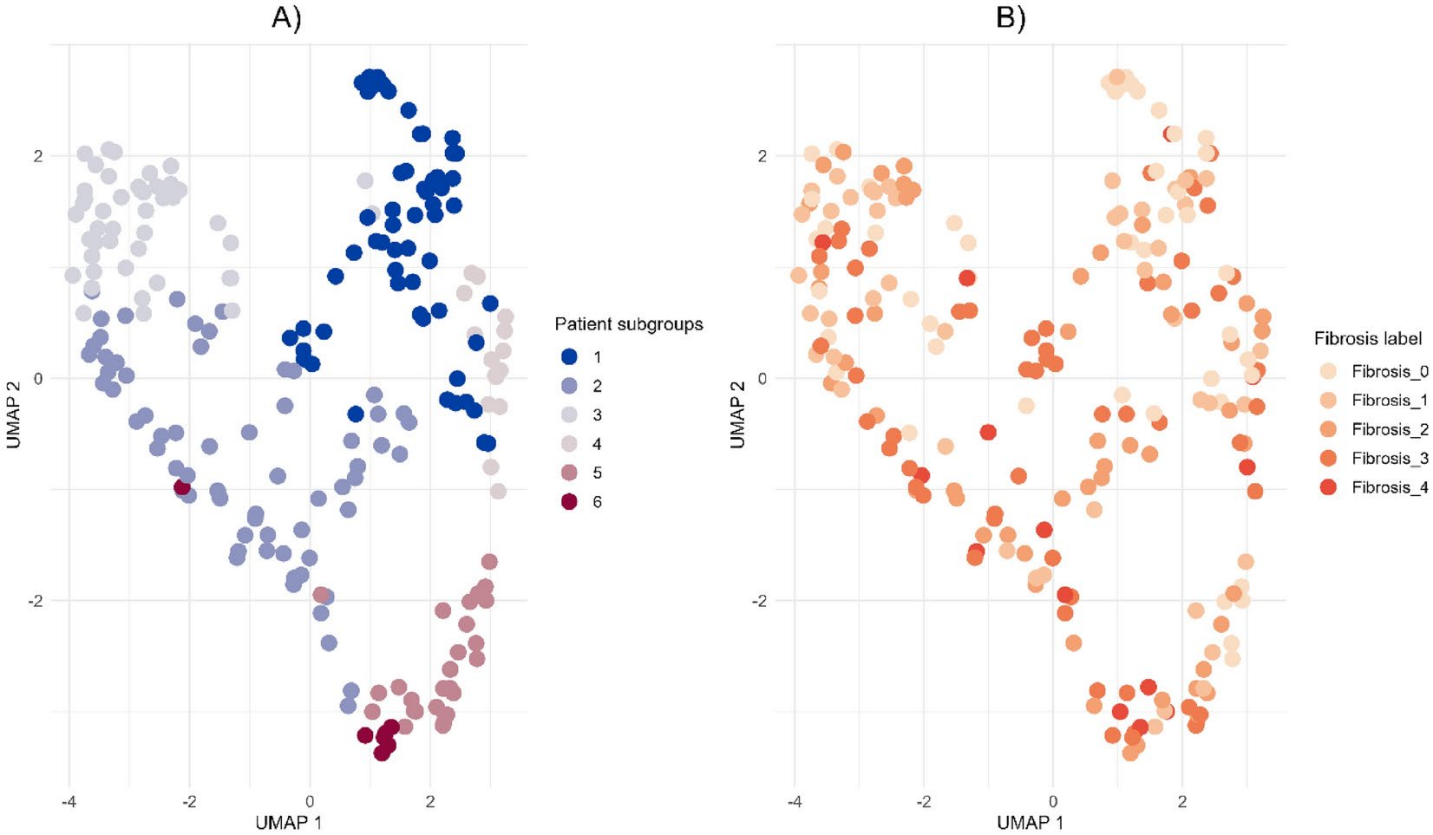
UMAP plot based on the clustered discovery dataset. (**A**) Colored by patient subgroup, (**B**) colored by fibrosis label. Subgroup 1 (n = #57), subgroup 2 (n = #64), subgroup 3 (n = #46), subgroup 4 (n = #15), subgroup 5 (n = #27) and subgroup 6 (n = #7).

**Fig. 6:**
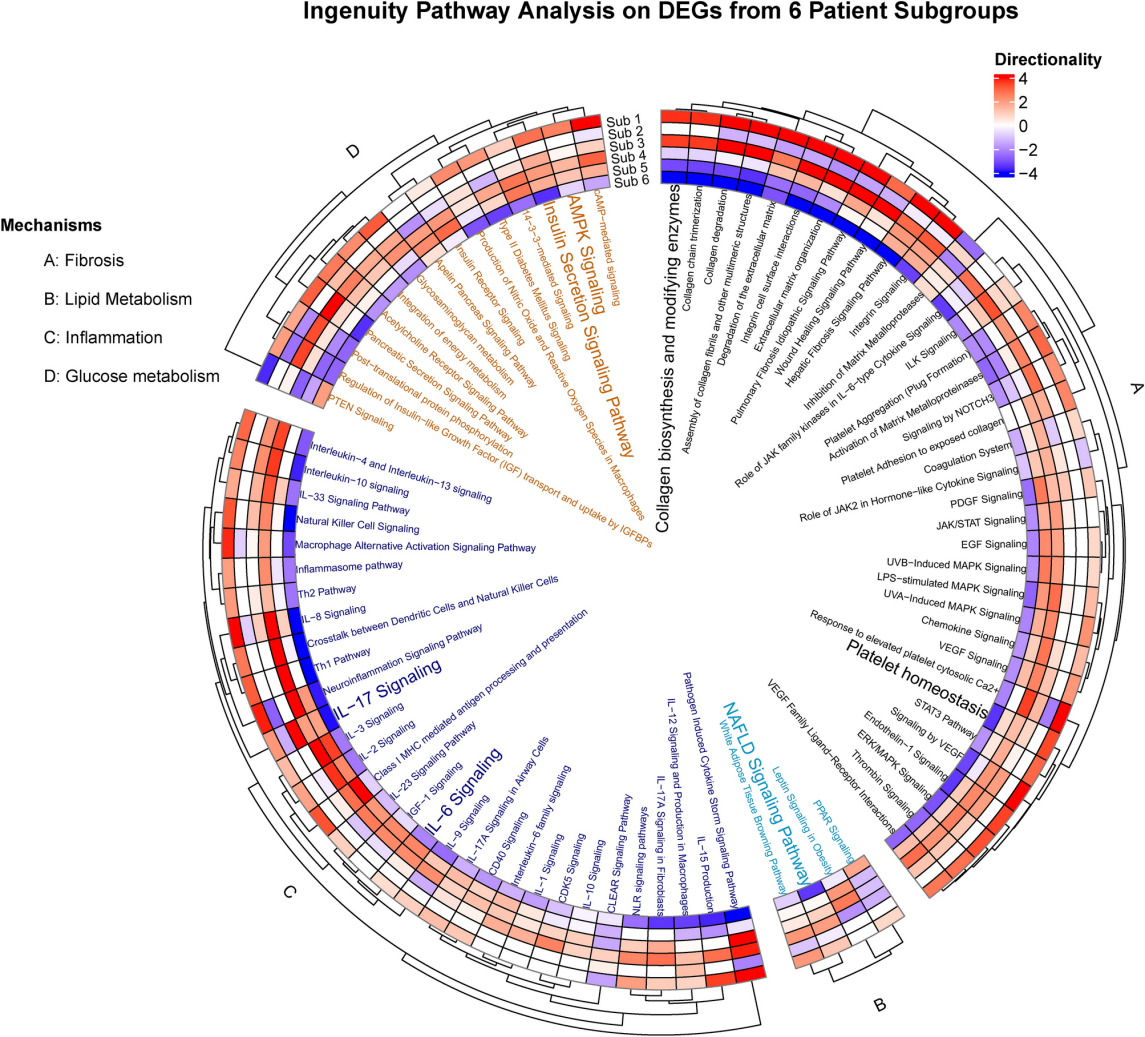
Canonical pathways in patient subgroups. DEGs from one versus rest DESeq2 analysis were analyzed using Ingenuity Pathway Analysis. A manually selected list of relevant canonical pathways in fibrosis pathology was used. Colors indicate directionality Z score, where a higher enrichment indicates higher value.

**Fig. 7:**
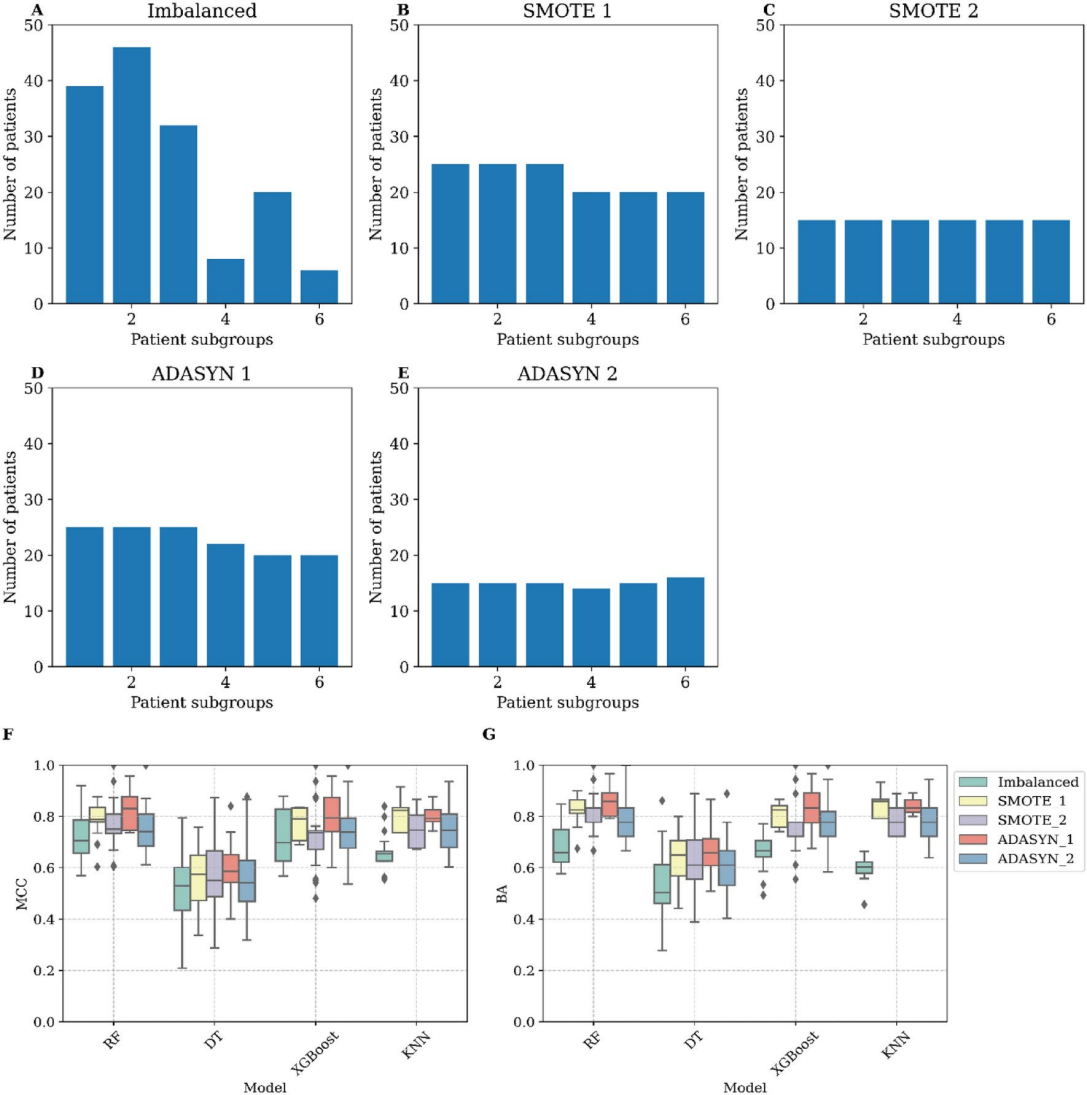
Data Augmentation and Hyperparameter Optimization. This figure illustrates the impact of data augmentation techniques on training input datasets with varying patient subgroup sizes (**A**–**E**) and evaluates the performance of hyperparameter optimization metrics across four machine learning algorithms (**F**, **G**). The datasets include the original imbalanced dataset (**A**) and augmented datasets generated using SMOTE-1 (**B**), SMOTE-2 (**C**), ADASYN-1 (**D**), and ADASYN-2 (**E**). Hyperparameter optimization was conducted for Random Forest, Decision Trees, XGBoost, and k-Nearest Neighbors. Performance was assessed with Matthews Correlation Coefficient (MCC) and Balanced Accuracy (BA). Evaluation employed nested cross-validation with stratified inner (k = 2) and outer (n = 5) fold cross-validation, using metrics obtained from 50 iterations per model using the randomsearch() implementation. SMOTE-1 and ADASYN-1 adjusted training split subgroup sizes to (Subgroup 1 = 25, Subgroup 2 = 25, Subgroup 3 = 25, Subgroup 4 = 20, Subgroup 5 = 20, Subgroup 6 = 20), while SMOTE-2 and ADASYN-2 adjusted them to (Subgroup 1 = 15, Subgroup 2 = 15, Subgroup 3 = 15, Subgroup 4 = 15, Subgroup 5 = 15, Subgroup 6 = 15).

**Fig. 8:**
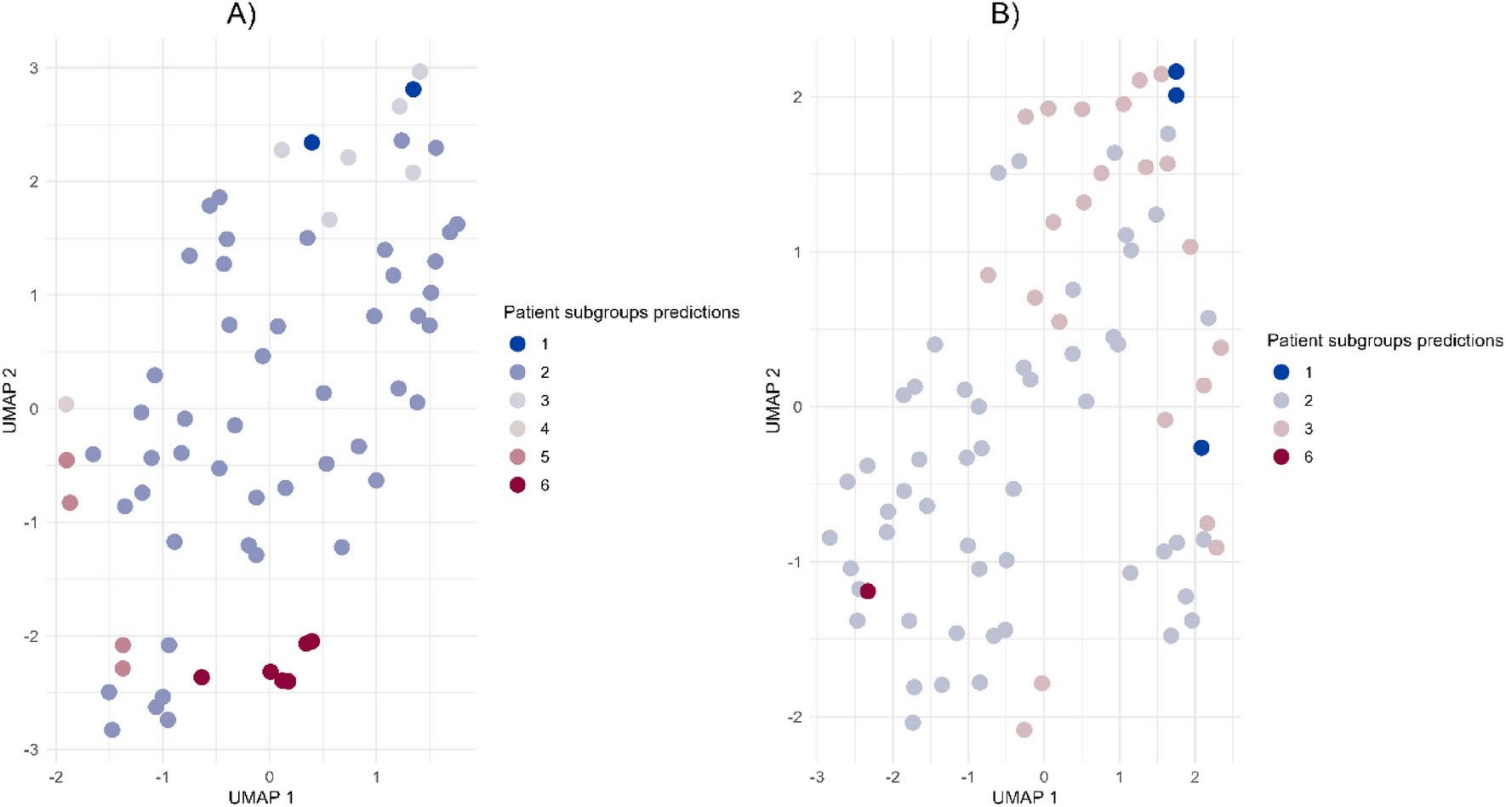
Patient subgroup predictions in the unseen dataset using the 14 hub gene space from the discovery dataset. A) Patient subgroups predictions in the FFPE dataset. Subgroup 1 (n = #2), subgroup 2 (n = #48), subgroup 3 (n = #6), subgroup 4 (n = #1), subgroup 5 (n = #4) and subgroup 6 (n = #6). B) Patient subgroups predictions in the Hoang dataset. Subgroup 1 (n = #3), subgroup 2 (n = #52), subgroup 3 (n = #22) and subgroup 6 (n = #1).

Furthermore, other modules depict disease-related pathways such as stress-related signals in module 12 with EIF signaling pathway and upstream regulator RICTOR. Interestingly, AMPK signaling (modules 6 and 10) may be of interest in the context of MASLD. In addition to the fibrosis signaling processes, SP1 transcription factor and VEGF growth factor are relevant in both modules 4 and 7, therefore overlapping with canonical fibrosis signaling. To investigate the directionality in fibrosis gene modules the genes from module 4 (448 genes) and module 7 (260 genes) were compared to the DEGs (F score 4 vs F0, 247 genes) shared in the discovery dataset (Govaere) and other datasets (Hoang and FFPE). The DEGs lists from the three datasets were compared with the genes in gene module 4 and 7. Interestingly, various upregulated DEGs were present in module 4 (e.g. *COL1A1, PLVAP, PAPLN, LAMC3*) and module 7 (e.g. *THY1, AEBP1, EPCAM, ITGBL1, EFEMP1, CFTR, SOX9, LOXL4*)  (Fig. 4). This was predominantly visible in module 7 as module 4 overlaps with immune processes.All patients were visualized in a UMAP plot using the 14 hub genes from the discovery dataset to evaluate whether the patient population in the discovery dataset forms specific patient subgroups. The plot shows the six patient subgroups separation and their respective fibrosis label distribution in a 2D space (Fig. 5). In addition, to identify the biological patterns in the six patient subgroups, differential expression analysis was performed with a one versus rest approach (e.g. subgroup 1 vs all). This resulted in subgroup-specific DEGs, which were mapped to the canonical pathways from IPA analysis (See Fig. 6). These showed distinct patterns in various key fibrotic mechanisms. For example, collagen biosynthesis is upregulated in subgroup 1 and 3, while downregulated in subgroup 5 and 6. Additionally, other relevant mechanisms such as cytokine signaling (e.g. IL-6, IL-17 signaling), MAFLD (NAFLD) signaling, platelet homeostasis, insulin secretion signaling and AMPK signaling show distinct patterns across patient subgroups.Since the identification of the 14 gene modules was based on many genes (~11 K genes), a classification model with most relevant features (model-based feature selection) was trained to predict the six patient subgroups. Therefore, several prediction models were tested and generated to classify the six patient subgroups. Since the actual group sizes of the patient subgroups is imbalanced (Fig. 7A), we used over-under sampling strategies (Fig. 7B, C, D and E) to optimize the datasets and generalizability of the models. Models were evaluated based on Matthew’s correlation coefficient (Fig. 7F) and balanced accuracy (Fig. 7G). Five different training input datasets (Imbalanced, SMOTE-1, SMOTE-2, ADASYN-1 and ADASYN-2) and 4 algorithms (random forest, decision trees, xgboost, and k-nearest neighbors) were used for hyperparameter optimization with a randomsearch() implementation. The random forest algorithm and ADASYN-1 training set were selected (See supplementary Tables 1 and 2) based on metrics and non-parametric paired group comparisons (See supplementary tables). For further model hyperparameter optimization, random forest and ADASYN-1 training set were used with a gridsearch() implementation. The final model with the best validation metrics was tested on the 30% test set, which showed a balanced accuracy above 80%. (See Fig. 7). Using the final model, two unseen datasets were classified into the six patient subgroups and visualized in a UMAP using the 14 hub genes identified in the discovery dataset. Patient subgroups in unseen datasets showed separation (See Fig. 8).

## Discussion

We investigated the hepatic expression patterns in a large population to obtain mechanistic insight into biological complexity and stratify a patient population of MASLD-MASH into patient subgroups. MASLD disease and comorbidities are increasing hepatic complications worldwide with a significant health burden and long-term consequences. Currently, there is only one available FDA approved drug (Resmetirom recently accepted) possibly linked to the absence of a precise patient stratification^33^. Our work shows a potential alternative for patient population stratification into patient subgroups based on commonalities in gene expression in patients using hub genes signature of representative MASLD-MASH mechanisms. Additionally, patient subgroups were characterized on the gene and pathway level to further pinpoint potential therapies to combat MASLD-MASH manifestations.

First, to obtain mechanistic insight, a WGCNA analysis on a large discovery dataset allowed us to identify 14 gene modules. These gene modules represent pathogenesis-related molecular mechanisms and biological complexity in individuals with varying degree of biopsy-diagnosed fibrosis and steatosis. Of note, on the MASLD-MASH continuum spectrum gene modules were linked to multiple well-known key mechanisms including hepatic fibrosis^3^, inflammation^2^, cholesterol biosynthesis^34^, as well as to recently associated MASLD-related mechanisms such as AMPK signaling^35^. Hepatic fibrosis related genes were distributed in both gene module 4 (COL4A2, COL1A1 and TIMP1) and gene module 7 with the involvement of transcription factor AEBP1 and downstream genes (EFEMP1, ITGBL1, LAMC3). Of note, transcription factor AEBP1 was upregulated in F4 advanced fibrosis (see Fig. 4) and has been suggested as a potential drug target candidate previously^36,37^. Altogether, this might indicate that multiple mechanisms in different modules drive patient population heterogeneity, biological complexity, and have a mechanistic link to fibrosis pathogenesis.

Secondly, considering the biological heterogeneity embedded in the 14 modules their corresponding 14 hub gene signature was used to stratify the discovery dataset into six patient subgroups and provide a more realistic patient stratification. Of note, the six patient subgroups remained separate in the 11k gene space used to identify the gene modules. This suggests a high regulatory property in the hub gene signature and patient subgroup separation. In support of our stratification methodology, different approaches using clustering methods have been used to stratify patient populations to circumvent the biological complexity and heterogeneity to find distinct pathology patterns in patient subgroups^7,23–26^.

Thirdly, to characterize distinct pathology patterns and delve into the pathological subtype manifestations between patient subgroups, subgroup-specific DEGs (e.g. Subgroup 1 vs Subgroup 2–6) were identified using differential expression analysis. Of relevance, subgroup-specific DEGs were mapped to IPA pathways and showed differentially expressed canonical pathways. For instance, directionality is opposite between subgroup 1 and subgroup 6 in both extracellular matrix organization and integrin signaling pathway, suggesting different degree of pathology across subgroups. Recently, a similar approach, using the proteome signature of inflammatory serum proteins (e.g. IL6, IL18), patient subgroups with distinct biology were identified, for instance showing differences in cytokine signaling patterns between MASLD and MASH (tendency for lower Interleukin-6 in MASH)^7^. In line with our results, subgroup 6 (highest average pathology scores) showed downregulation of Interleukin-6 signaling. However, relationship between pathology degree and IL-6 signaling may depend on other factors (e.g. visceral adiposity, body mass index)^8,9^. Collectively, these distinct pathology patterns in patient subgroups may contribute to the complexity in liver disease manifestations and possibly suggest their consideration to achieve a successful pharmacotherapy. Finally, the final classification model allowed us to predict the different six subgroups in two unseen datasets (smaller in population size with mild-moderate fibrosis stages individuals) showing separation in the 14 hub gene signature space.

## Limitations

These findings should be interpreted with caution in the context of fibrosis development heterogeneity and the relationship of biological patterns with temporality in the MASLD-MASH continuum. Since the patient population was stratified only using their liver transcriptome on a single point in time, it was not possible to capture hepatic expression dynamics. Moreover, this study lacks the access to more metadata as well as other data from other omics technologies (e.g. proteome, microbiome). In this regard, future studies considering genetics (SNPs), epigenetic factors as well as relationship with clinical phenotypes and metadata may improve stratification with a higher fidelity to capture patient variability.

## Conclusion

Our work shows a potential alternative for patient population stratification based on hub gene signature of representative MASLD-MASH mechanisms. We have shown that different gene modules drive patient heterogeneity which also have a mechanistic link to pathological fibrosis. These findings hold significant implications for patient stratification in clinical trials assessing potential pharmacotherapies. Moreover, the findings can be used for patient subgroup-specific consideration in the selection and validation of preclinical models for novel target discovery and therapeutic intervention design. Future research is needed to validate the relationship of the subgroup-specific pathway patterns and identify novel protein targets for virtual screening and/or in vitro validation in preclinical models.

## Supplementary Information

Supplementary Tables.

## Data Availability

The datasets used and/or analyzed during the current study are available from the respective GEO repository. Code for the data analysis on this article is available in (https://github.com/mangonzalez12/Mechanistic-Pathology-NAFLD.git).
