# Supplementary material for: Identifying patient subgroups in MASLD and MASH-associated fibrosis: molecular profiles and implications for drug development
Source: Sci Rep. 2024 Oct 7;14:23362. doi: 10.1038/s41598-024-74098-w (PMC11458909; doi:10.1038/s41598-024-74098-w)
Supplement: Supplementary file 1 — Supplementary Tables. [file 41598_2024_74098_MOESM1_ESM.pdf]

Supplementary data

Supplementary Table 1. Validation of training data types with machine learning (ML) methods. For each ML method, we evaluated five types of input data as described in the methods section: Data augmentation was applied to the training split (70%), using over-under sampling with SMOTE and ADASYN. The Mathews correlation coefficient (MCC), balanced accuracy, F1 score, Precision and Recall were used as the performance metrics resulting from the five-fold nested cross-validation repeated for ten times (5*10). For MCC, +1 indicates perfect prediction, 0 random prediction and –1 perfect inverse prediction. For Balanced accuracy (Bal Acc), 1 indicates perfectly accurate prediction. ML method abbreviations: DT, decision trees; KNN, k nearest neighbours; RF, random forest and XGBoost, extreme gradient boosting.

ML model [μ+ SD]	Data type	MCC[μ+ SD]	Bal_Acc [μ+ SD]	Precision [μ+ SD]	Recall [μ+ SD]
DT (0.57 + 0.09)	ADASYN_1	0.55 (0.13)	0.62 (0.11)	0.61 (0.11)	0.61 (0.11)
	ADASYN_2	0.54 (0.13)	0.60 (0.11)	0.60 (0.11)	0.60 (0.11)
	Imbalanced	0.46 (0.13)	0.47 (0.11)	0.58 (0.09)	0.58 (0.09)
	SMOTE_1	0.54 (0.10)	0.62 (0.08)	0.61 (0.08)	0.61 (0.08)
	SMOTE_2	0.58 (0.14)	0.63 (0.12)	0.63 (0.12)	0.63 (0.12)
KNN (0.72 + 0.09)	ADASYN_1	0.80 (0.04)	0.83 (0.03)	0.82 (0.03)	0.82 (0.03)
	ADASYN_2	0.76 (0.11)	0.78 (0.10)	0.79 (0.09)	0.79 (0.09)
	Imbalanced	0.67 (0.09)	0.59 (0.06)	0.74 (0.07)	0.74 (0.07)
	SMOTE_1	0.80 (0.06)	0.84 (0.05)	0.83 (0.05)	0.83 (0.05)
	SMOTE_2	0.76 (0.06)	0.8 (0.06)	0.8 (0.06)	0.8 (0.06)
	ADASYN_1	**0.81 (0.07)**	**0.85 (0.05)**	**0.84 (0.06)**	**0.84 (0.06)**

RF (0.93 + 0.10)	ADASYN_2	0.77 (0.12)	0.80 (0.10)	0.81 (0.10)	0.81 (0.10)
	Imbalanced	0.74 (0.09)	0.69 (0.08)	0.80 (0.07)	0.80 (0.07)
	SMOTE_1	0.78 (0.07)	0.82 (0.05)	0.81 (0.05)	0.81 (0.05)
	SMOTE_2	0.79 (0.12)	0.82 (0.10)	0.82 (0.10)	0.82 (0.10)
XGB (0.81 + 0.14)	ADASYN_1	0.80 (0.09)	0.83 (0.07)	0.82 (0.07)	0.82 (0.07)
	ADASYN_2	0.75 (0.12)	0.78 (0.11)	0.78 (0.11)	0.78 (0.11)
	Imbalanced	0.71 (0.10)	0.65 (0.06)	0.77 (0.08)	0.77 (0.08)
	SMOTE_1	0.77 (0.04)	0.81 (0.03)	0.80 (0.03)	0.80 (0.03)
	SMOTE_2	0.72 (0.12)	0.76 (0.10)	0.76 (0.10)	0.76 (0.10)

Supplementary Table 2. Wilcoxon test pair comparisons between the different types of input data with the support vector machine with Random Forest Classifier (RF). Comparisons are based on the Mathews correlation coefficient (MCC) values resulted from the five-fold nested crossed-validation repeated for ten times (5*10). Significant p-values were determined with a value cut-off of 0.05 after Benjamin-Hochberg (BH) correction.

**Dataset 1**	**Dataset 2**	**Wilcoxon p-value**
Imbalanced	SMOTE_1	0.15
Imbalanced	SMOTE_2	0.07
Imbalanced	ADASYN_1	**0.00**
Imbalanced	ADASYN_2	0.14
SMOTE_1	SMOTE_2	0.87
SMOTE_1	ADASYN_1	**0.01**
SMOTE_1	ADASYN_2	0.56
SMOTE_2	ADASYN_1	0.18
SMOTE_2	ADASYN_2	0.17
ADASYN_1	ADASYN_2	**0.02**
